# Long-term impact of the metabolic status on weight loss-induced health benefits

**DOI:** 10.1186/s12986-022-00660-w

**Published:** 2022-03-28

**Authors:** Dominik Soll, Julia Gawron, Laura Pletsch-Borba, Joachim Spranger, Knut Mai

**Affiliations:** 1 Department of Endocrinology and Metabolism, Charité – Universitätsmedizin Berlin, corporate member of Freie Universität Berlin, Humboldt-Universität Zu Berlin, and Berlin Institute of Health, Chariteplatz 1, 10117 Berlin, Germany; 2 Charité Center for Cardiovascular Research, Charité – Universitätsmedizin Berlin, corporate member of Freie Universität Berlin, Humboldt-Universität Zu Berlin, and Berlin Institute of Health, 10117 Berlin, Germany; 3 DZHK (German Centre for Cardiovascular Research), partner site Berlin, Berlin, Germany; 4 NutriAct-Competence Cluster Nutrition Research Berlin-Potsdam, Nuthetal, Germany

**Keywords:** Healthy obesity, Weight loss, Insulin sensitivity, Quality of life

## Abstract

**Background:**

While short-term effects of weight loss on quality of life and metabolic aspects appear to be different in metabolically healthy (MHO) and metabolically unhealthy obese (MUO), respective long-term data is still missing. Given the high relevance of long-term changes, we aimed to address these in this post-hoc analysis of the MAINTAIN trial.

**Methods:**

We analyzed 143 overweight/obese subjects (BMI ≥ 27 kg/m^2^, age ≥ 18 years) before and after a 3-month weight loss program (≥ 8% weight loss), after a 12-month period of a randomized weight maintenance intervention (n = 121), and after another 6 months without intervention (n = 112). Subjects were retrospectively grouped into MHO and MUO by the presence of metabolic syndrome and secondarily by estimates of insulin sensitivity (HOMA-IR and ISI_Clamp_). Quality of life (QoL), blood pressure, lipids, HOMA-IR, and ISI_Clamp_ were assessed and evaluated using mixed model analyses.

**Results:**

Despite similar short- and long-term weight loss, weight loss-induced improvement of HOMA-IR was more pronounced in MUO than MHO after 3 months (MHO: 2.4[95%-CI: 1.9–2.9] vs. 1.6[1.1–2.1], *p* = 0.004; MUO: 3.6[3.2–4.0] vs. 2.0[1.6–2.4], *p* < 0.001; *p* = 0.03 for inter-group comparison). After 21 months, the beneficial effect was no longer seen in MHO (2.0[1.5–2.6], *p* = 1.0), while it remained partially preserved in MUO (2.9[2.4–3.3], *p* = 0.002). QueryShort-term improvements of lipid parameters were similar in both groups. However, long-term improvements of HDL-cholesterol and triglycerides were only seen in MUO (44.4[41.5–47.4] vs. 49.3[46.2, 52.3] mg/dl, *p* < 0.001; 176.8[158.9–194.8] vs. 138.8[119.4–158.3] mg/dl, *p* < 0.001, respectively) but not in MHO. Weight loss-induced improvements in the QoL and particularly the physical health status were maintained in MUO until the end of the trial, while benefits disappeared over time in MHO. Group allocation by HOMA-IR and ISI_Clamp_ revealed higher benefits for MUO mainly in parameters of the glucose metabolism and QoL.

**Conclusions:**

Our data demonstrates stronger and longer-lasting improvements of metabolism and QoL in MUO after weight loss.

*Trial registration* (ClinicalTrials.gov): NCT00850629. Registered 25 February 2009, https://clinicaltrials.gov/ct2/show/NCT00850629.

**Supplementary Information:**

The online version contains supplementary material available at 10.1186/s12986-022-00660-w.

## Introduction

Obesity is a chronic, systemic, and multifactorial disease and is defined—according to the world health organization (WHO)—by a body mass index (BMI) of 30 kg/m^2^ or more. Despite its preventable nature, the prevalence of obesity has been increasing over the last decades [1]. Obesity is associated with several musculoskeletal, metabolic, and cardiovascular diseases, amongst others [2].

Weight loss has been shown to improve the obesity-related metabolic disturbances and is therefore recommended in the European guideline for the management of obesity, among others [3]. In general, positive effects on metabolic diseases, such as lower incidence of type 2 diabetes and increased insulin sensitivity, have been shown for a weight loss of 5–8% [4, 5]. Given the rising number of obese subjects and limited resources, the identification of subjects who will benefit most from weight loss interventions may help to optimize current treatment strategies.

More than 15 years ago, a subset of obese patients has been identified that is not affected by the mentioned metabolic changes that frequently go along with obesity. Primarily, they remain insulin sensitive despite being obese [6]. Current data indicates that acute metabolic benefits of weight loss were more pronounced [7] or did only occur in metabolically unhealthy obese (MUO) [8, 9], but not in metabolically healthy obese (MHO). Although this data supports a metabolic improvement by dietary weight loss primarily in MUO, long-term data on the different or comparable effectiveness of temporary lifestyle interventions for MHO and MUO is not yet available. This is crucial as the impact of temporary lifestyle interventions on weight loss is often frustrating due to frequently observed weight regain [10].

Although the classification of MHO and MUO is frequently based on the presence or absence of the metabolic syndrome [7, 11, 12], this still remains inconsistent and is a matter of debate. The International Diabetes Federation (IDF) defines the metabolic syndrome as a specific cluster of medical conditions with central obesity, insulin resistance, hypertension, and dyslipidemia [13]. Nevertheless, the presence of insulin resistance in fasting state or during an oral glucose tolerance test, or the results of a hyperinsulinemic-euglycemic clamp are also used to define metabolic health [7, 8, 14, 15]. The added value of these definitions of metabolic health is currently still unknown.

Given this lack of evidence, we report short- and long-term data from a 21-month weight loss/weight maintenance trial focusing on metabolic healthy and unhealthy obese. In detail, we intend to elucidate whether a lifestyle-based weight loss intervention demonstrates different efficacies regarding body weight reduction, metabolic improvement, and quality of life (QoL) in obese subjects differing in their metabolic health status. The efficacy was especially assessed in the long term. In order to do so, we used the IDF criteria from 2005 for metabolic syndrome to separate MUO from MHO individuals as they are well-established and easily applicable for primary and special health care providers in clinical practice. To evaluate the impact of alternative definitions of metabolic health, we performed comparable analyses after classification of MUO and MHO using the HOMA-IR and the hyperinsulinemic-euglycemic clamp as an easily estimable and a more precise determinant of insulin resistance, respectively.

## Study design and methods

### Participants and study design

The study (MAINTAIN trial, ClinicalTrials.gov registry number NCT00850629) was performed between 2010 and 2016 in Berlin, Germany. Detailed trial information has been reported previously [10, 16, 17]. The flow of participants is shown in Additional file 1: Figure S1. In short, 156 overweight and obese subjects (BMI ≥ 27 kg/m^2^, age ≥ 18 years) underwent a 3-month-weight loss program, realized by caloric restriction using a very low energy diet for 8 weeks (replacement of all meals by a formula diet with 800 kcal per day) and an energy-reduced diet (healthy food diet with approximately 1500 kcal per day) for the following 4 weeks, both accompanied by nutritional counseling, physical exercises, and psychological advice. Subjects that experienced a prior weight loss of more than 5 kg in the last 2 months, changed their smoking habits or diets within the last 3 months, were pregnant, or suffered from other endocrine disorders, eating disorders, or severe chronic diseases were not included in this trial. All participants who succeeded to lose at least 8% of their body weight (n = 143) were then randomized into an intervention or control group for 12 months of weight maintenance. The intervention comprised a continuous multimodal counseling focusing on caloric restriction, nutritional counseling, physical exercises, and psychological support. In contrast, the control group was under free living conditions without further counseling. Following the randomized intervention period, both groups underwent a 6-month-follow-up period without any further intervention. More details regarding the lifestyle intervention can be found in the Additional file 1.

Retrospectively, all randomized subjects were allocated to be MHO or MUO depending on the absence or presence of metabolic disturbances. In this regard, three different definitions were used. First, subjects were categorized in accordance to the criteria of the metabolic syndrome as defined by the IDF [18]: central obesity defined as waist circumference ≥ 94 cm for men and ≥ 80 cm for women plus any two of the following four factors: (1) elevated triglycerides level > 150 mg/dl or intake of lipid-lowering drugs, (2) reduced HDL cholesterol < 40 mg/dl for men and < 50 mg/dl for women, (3) raised blood pressure ≥ 130 mmHg for systolic or ≥ 85 mmHg for diastolic blood pressure or intake of antihypertensive drugs, (4) raised fasting plasma glucose ≥ 100 mg/dl or previously diagnosed type 2 diabetes. Next, subjects were allocated to be MHO/MUO by their respective HOMA-IR before weight loss. Here, subjects with a HOMA-IR ≥ median HOMA-IR (~ 2.19) were distributed to MUO, subjects with a HOMA-IR < median HOMA-IR to MHO. Last, subjects were allocated by their respective insulin sensitivity index (ISI_Clamp_) before weight loss. Here, subjects with an ISI_Clamp_ ≥ median ISI_Clamp_ (~ 0.058 mg kg^−1^ min^−1^/(mU l^−1^)) were defined as MHO, subjects with a ISI_Clamp_ < median ISI_Clamp_ were classified as MUO. Due to the lack of reliable cut-offs for a “pathological” HOMA-IR or ISI_Clamp_, the group was divided by the respective median.

### Procedures

Fasting blood sampling as well as measurements of blood pressure, waist circumference, body weight, height, and assessment of QoL were performed before (T_-3_) and after (T_0_) weight loss, 12 months (T_12_) after randomization and after another 6 months without active intervention (T_18_). At T_-3_, T_0_, and T_12_, subjects underwent a hyperinsulinemic-euglycemic clamp.

Blood samples were centrifuged; plasma and serum samples were frozen immediately at − 80 °C. Glucose was measured using the glucose oxidase method (Dr. Müller Super GL, Freital, Germany). HbA1c was measured by high-performance liquid chromatography using the VARIANT II (Bio-Rad, Hercules, US). Lipids were measured by standard laboratory methods using Cobas ISE direct and c111 Analyzer (Roche Diagnostics, Mannheim, Germany). Serum insulin was measured by fluoroimmunometric assay (AutoDelfia; Perkin Elmer, Rodgau, Germany). Fat mass was assessed by bioelectrical impedance analysis using the AKERN BIA 101 (SMT medical GmbH & Co. KG, Würzburg, Germany).

In order to assess potential changes in quality of life and patient-reported health status that might accompany weight loss and maintenance, participants were asked to fill in the Short Form (36) Health Survey (SF-36). It contains 36 items in eight subscale scores: general health perceptions, physical functioning, role limitations due to physical problems, bodily pain, mental health, role limitations due to emotional problems, vitality, and social functioning. A physical component summary score (PCS) is derived from the first four, a mental component summary score (MCS) from the latter four components [19].

The insulin resistance index HOMA-IR was calculated as previously described: fasting glucose (mg/dl) × fasting insulin (mU/l)/405 [20].

The hyperinsulinemic-euglycemic clamp involved infusion of insulin at the rate of 40 mU per m^2^ of body surface per minute and of glucose at an individual rate to fix plasma glucose levels at 80 mg/dl ± 8 mg/dl. The steady state was defined as plasma glucose levels of 80 mg/dl ± 8 mg/dl for at least 30 min. The ISI_Clamp_ was calculated by dividing the glucose infusion rate at steady state (mg/min) per body weight (kg) by plasma insulin at steady state (mU/l) [20].

### Statistical analysis

Data of 143 subjects who lost at least 8% of their initial body weight was analyzed. The primary analysis was performed using group allocation according to IDF criteria. Statistical analysis was performed using RStudio Version 1.2.1335 (RStudio Inc., Boston, MA) and the R software package. We performed a linear mixed effects analysis of the relationship between anthropometric/metabolic parameters and time using the *nlme* package. For group comparisons, additional analysis of the relationship between anthropometric/metabolic parameters and the interaction of time and group affiliation was done. We entered age, sex, and randomization state (intervention group or control group) as fixed effects and potential confounders. In order to adjust for BMI change in the course of the study, we repeated this analysis after additional inclusion of either BMI, waist circumference, or body fat mass at all time-points as a fixed effect. Intercepts for subjects were considered as a random effect. Results were considered to be significant, if the two-sided α was below 0.05. Presented data represents estimated marginal mean and 95%-confidence interval (CI). *P* values were obtained from comparisons using the *emmeans* package. Adjustment for multiple testing was performed by Bonferroni correction.

## Results

### Clinical and metabolic characteristics at baseline

General data on the trial population has already been published previously [10, 16, 21]. Respective relevant data can be found in Additional file 1: Table S1. To eliminate the effect of interfering factors, age, sex, and randomization state were included as fixed effects in the mixed effect model analysis for group comparisons. Using IDF criteria, there were significant differences between both MHO and MUO at T_-3_ for waist circumference (Δ(MHO-MUO) − 6.5 [95% CI: − 10.4, − 2.6] cm, *p* = 0.001) and BMI (− 2.2 [− 4.2, − 0.2] kg/m^2^, *p* = 0.03). Consistent with the parameters for allocation, both groups also differed in triacylglycerol levels (− 57.7 [− 84.3, − 31.1] mg/dl, *p* < 0.001), HDL cholesterol (11.1 [6.8, 15.3] mg/dl, *p* < 0.001), fasting glucose (− 12.6 [− 16.9, − 8.2] mg/dl, *p* < 0.001), and HbA1c (− 0.42 [− 0.65, − 0.18], *p* < 0.001). Additionally, MHO and MUO differed in the estimates for insulin resistance HOMA-IR (− 1.3 [− 1.9, − 0.7], *p* < 0.001) and ISI_Clamp_ (0.03 [0.02, 0.04] mg kg^−1^ min^−1^/(mU l^−1^), *p* < 0.001), but in none of the other presented parameters. (Table 1).

**Table 1:** Estimates of clinical parameters of MHO/MUO following the IDF definition of metabolic syndrome

	Metabolically Healthy Obese (MHO)				Metabolically Unhealthy Obese (MUO)				*p*-value (MHO vs MUO)		
	T_-3_	T_0_	T_12_	T_18_	T_-3_	T_0_	T_12_	T_18_	T_-3_ → T_0_	T_-3_ → T_12_	T_-3_ → T_18_
n: m/w	59: 9/50	59: 9/50	46: 7/39	44: 6/38	84: 22/62	84: 22/62	75: 19/56	68: 19/49			
Randomization: control/intervention	29/30				42/42						
Age [years]	48.6 [45.2,52.0]				51.9 [49.2,54.5]						
BMI [kg/m2]	36.0 [34.2,37.7]	31.4 [29.7,33.1]***	32.5 [30.8,34.3]***	33.5 [31.7,35.2]***	38.2 [36.8,39.6]	33.5 [32.1,34.9]***	34.1 [32.7,35.6]***	35.2 [33.8,36.6]***	1	1	1
Waist circumference [cm]	107.6 [104.2,110.9]	99.4 [96,102.7]***	100.9 [97.5,104.4]***	101 [97.5,104.5]***	114 [111.3,116.8]	103.8 [101.1,106.5]***	104.4 [101.6,107.2]***	106 [103.2,108.8]***	0.6	0.14	1
Serum glucose [mg/dl]	82.4 [78.8,86]	80.8 [77.2,84.4]	84.3 [80.4,88.2]	86.9 [83.0,90.9]	95.0 [92.0,97.9]	86.1 [83.1,89.0]***	89.7 [86.6,92.8]**	93.2 [90.1,96.3]	0.01	0.02	0.06
HbA1c [%]	5.5 [5.3,5.7]	5.9 [5.7,6]**	5.4 [5.2,5.6]	5.5 [5.3,5.7]	5.9 [5.8,6.1]	5.9 [5.8,6.1]	5.6 [5.4,5.8]***	5.7 [5.6,5.9]	0.02	0.66	1
HOMA-IR	2.4 [1.9,2.9]	1.6 [1.1,2.1]**	1.9 [1.3,2.4]	2.0 [1.5,2.6]	3.6 [3.2,4]	2.0 [1.6,2.4]***	2.3 [1.9,2.7]***	2.9 [2.4,3.3]**	0.03	0.06	0.91
ISI_Clamp_ [mg kg^−1^ min^−1^/(mU l^−1^)]	0.08 [0.07,0.09]	0.10 [0.09,0.11]***	0.09 [0.08,0.10]*	-	0.05 [0.04,0.06]	0.08 [0.07,0.09]***	0.07 [0.07,0.08]***	-	0.31	0.23	-
Total cholesterol [mg/dl]	200.4 [190,210.9]	171.2 [160.8,181.6]***	192.5 [181.4,203.7]	192.4 [181.3,203.6]	198.1 [189.7,206.5]	167.5 [159.1,175.9]***	188.8 [180.1,197.5]*	191 [182.1,199.8]	1	1	1
LDL-Cholesterol [mg/dl]	121.2 [112.2,130.2]	102.1 [93.1,111.1]***	114.6 [105,124.1]	117.2 [107.6,126.7]	121.8 [114.5,129]	103.4 [96.1,110.6]***	113.7 [106.2,121.2]*	118.4 [110.8,126]	1	1	1
HDL-Cholesterol [mg/dl]	55.5 [51.9,59.2]	49 [45.3,52.6]***	58 [54.2,61.8]	58 [54.2,61.8]	44.4 [41.5,47.4]	41.8 [38.9,44.7]*	49.9 [46.9,52.9]***	49.3 [46.2,52.3]***	0.06	0.43	0.99
Triglycerides [mg/dl]	119.1 [97.0,141.2]	96.1 [74.1,118.2]	95.5 [70.9,120.0]	101.9 [77.3,126.5]	176.8 [158.9,194.8]	110.1 [92.2,128.1]***	149.1 [130.2,168.1]*	138.8 [119.4,158.3]***	0.01	1	1
Systolic RR [mmHg]	127.6 [122.4,132.8]	118.7 [113.6,123.9]*	121 [115.2,126.7]	121 [115.0,126.9]	132.4 [128.0,136.7]	121.4 [117.1,125.6]***	125 [120.5,129.5]	127.6 [122.9,132.3]	1	1	1
Diastolic RR [mmHg]	80.6 [77.8,83.4]	74.1 [71.2,76.9]***	77.9 [74.8,80.9]	79.8 [76.6,82.9]	80.5 [78.1,82.8]	74.8 [72.5,77.2]***	79 [76.6,81.4]	78.1 [75.6,80.6]	1	1	1

Values shown (except for n, randomization, and age) are estimated marginal means with 95% confidence intervals from model with adjustment to sex, age, and randomization

^*^ *p* < 0.05, ** *p* < 0.01, *** *p* < 0.001 vs. T_-3_ in respective group

### Short-term effects of weight loss on metabolism

During the initial 3-month weight reduction period, weight loss was similar in MHO and MUO (− 4.6 [− 5.4, − 3.7] kg/m^2^ vs. − 4.7 [− 5.4, − 4.0] kg/m^2^, *p* = 1.0). However, triglycerides and fasting glucose declined only in MUO, but not in MHO. (Table 1) While HOMA-IR was significantly more reduced in MUO than in MHO (− 1.7 [− 2.2, − 1.1] vs. − 0.8 [− 1.4, − 0.2], *p* = 0.03), HDL cholesterol was less modified by weight loss in MUO (− 2.7 [− 5.2, − 0.1] vs. -6.6 [− 9.6, − 3.5] mg/dl, *p* = 0.06), but the difference did not reach significance. In contrast, comparable improvements were detected in both groups in ISI_Clamp_, total cholesterol, and LDL cholesterol, as well as systolic and diastolic blood pressure.

Overall, group allocation based on HOMA-IR showed a stronger improvement in MUO only regarding fasting glucose and HOMA-IR (which was not modified in MHO) but not in lipids during the weight loss period (Additional file 1: Table S2). Comparably, group allocation according to ISI_Clamp_ revealed almost similar findings with different developments between groups only in HbA1c and HOMA-IR (Additional file 1: Table S3).

### Long-term effects of weight loss on metabolism

After initial weight loss, participants showed a moderate increase in BMI, but still maintained a significantly lower body weight until the end of the study after 18 months. There was no difference in the weight course between both, MUO and MHO (Table 1). In analogy, trajectory of waist circumference—as an established estimate of visceral obesity—was equal in both groups. Similarly, changes of ISI_Clamp_ were comparable in MUO and MHO. In contrast, in comparison to baseline MUO demonstrated improved HOMA-IR, triglycerides, and HDL cholesterol for up to 18 months and improved fasting glucose, total cholesterol, and LDL cholesterol until 12 months after weight loss (Table 1). However, these parameters were no longer improved in MHO at T_12_ or T_18_ compared to baseline. Nevertheless, between-group comparisons for ΔT_3_T_18_ did not reveal any significant differences in these parameters.

Group allocation depending on HOMA-IR revealed a long-term improvement of HOMA-IR in MUO but not in MHO (*p* < 0.001 for inter-group comparison) (Additional file 1: Table S2). A similar development of HOMA-IR in both groups is found if groups are separated by ISI_Clamp_ (Additional file 1: Table S3). However, this classification revealed additional differences in the long-term courses of BMI and ISI_Clamp_ between both groups.

### Improved quality of life persists in MUO but not in MHO

**Fig. 1:**
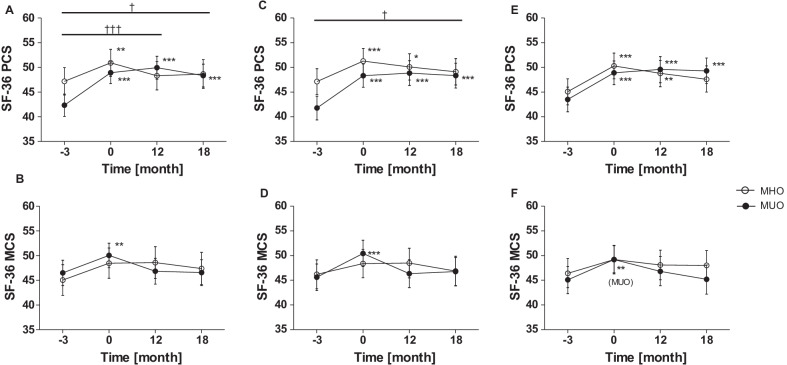
Effect of weight loss and maintenance on patient-reported quality of life in MHO/MUO. **A**, **B** MHO + MUO were assigned following the IDF criteria for metabolic syndrome. **C**, **D** MHO + MUO were assigned on the basis of HOMA-IR. **E**, **F** MHO + MUO were assigned on the basis of ISI_Clamp_. Values represent estimated marginal means with 95% confidence intervals from model with adjustment to sex, age, and randomization. *p < 0.05, **p < 0.01, ***p < 0.001 vs. T_-3_ in respective group. Where indicated, †/††/††† describe between-group comparisons, respectively. *SF-36* Short Form (36) Health Survey, *PCS* physical component summary score, *MCS* mental component summary score

At baseline, MHO had a higher physical health status than MUO (Δ(MHO-MUO) 4.8 [− 0.5, 10.2], *p* = 0.005), while mental health status did not differ (− 1.5 [− 7.5, 4.6], *p* = 0.4). After initial weight loss, the MUO reported an improved physical and mental health status, while the MHO indicated an improved physical but not mental health (Fig. 1A, B). Physical health status remained improved in MUO in the long term while the improvement of mental health disappeared after 12 months. In contrast, the short-term increase of physical health in MHO was already reversed after 12 months (Fig. 1A, B).

When groups are separated by HOMA-IR, the results regarding quality of life are almost similar (Fig. 1C, D), although improvement of physical health persisted up to 12 months after weight loss in MHO. Comparable changes and differences were found for group allocation by ISI_Clamp_ (Fig. 1E, F).

### Improved insulin resistance in MUO is not exclusively explained by weight loss

**Fig. 2:**
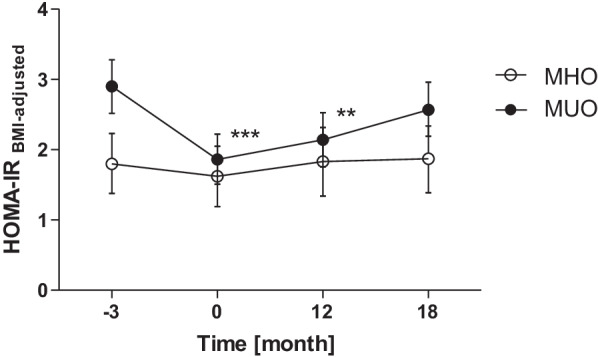
Effect of weight loss and maintenance on insulin resistance (adjusted for BMI) in MHO/MUO. MHO + MUO were assigned following the IDF criteria for metabolic syndrome. Values represent estimated marginal means with 95% confidence intervals from model with adjustment to sex, age, randomization, and BMI at all time-points. * *p* < 0.05, ** *p* < 0.01, *** *p* < 0.001 vs. T_-3_ in respective group

Considering the differential development of insulin resistance in MHO and MUO despite comparable weight loss, we aimed to further investigate the interrelation of improved obesity and insulin resistance. Hence, to eliminate the impact of body weight changes, we additionally adjusted the improvement of HOMA-IR for BMI. Interestingly, while the MHO did not present changes in HOMA-IR_BMI-adjusted_ anymore, it still was significantly declined by weight loss in MUO in the short- and medium-term (Fig. 2). Likewise, adjustment for waist circumference or body fat mass gave comparable results (Additional file 1: Figure S2).

## Discussion

In the presented study, we aimed to answer the question whether MHO and MUO subjects gain metabolic and health benefits to different extents from weight loss/weight maintenance by a multimodal lifestyle intervention. There are few studies that already addressed this issue in a short-term context, but were partially not consistent in their results [7–9, 11, 12, 15]. On the one hand, Dalzill et al*.* and Ruiz et al*.* found a similar improvement in HOMA-IR and several other metabolic parameters in MUO and MHO investigating 124 patients before and after a 9-month intensive lifestyle modification program and 78 postmenopausal women before and after a 3-month energy-restricted treatment, respectively [11, 12]. On the other hand, several previous studies described beneficial effects preferentially for MUO. While Janiszewski et al*.* studied 106 adults before and after various 3-to-6-month lifestyle interventions and found a larger increase of insulin sensitivity in MUO after weight loss [7], Kantartzis and colleagues demonstrated improved HOMA-IR and glucose levels during an OGTT only in MUO, but not in MHO in a report on 103 patients before and after a 9-month lifestyle intervention [8]. Similarly, Karelis et al*.*, who performed hyperinsulinemic-euglycemic clamps and reported data from 44 patients before and after a 6-month dietary intervention, described enhanced myocellular insulin sensitivity in MUO, but not in MHO[9]. Similar to our study, most previous studies reported data from secondary analyses. While weight loss ranged from 3 to 9% in the aforementioned studies, our patients lost more than 12% during weight loss. The patients’ age ranged from 45 to 60 years in most previous studies, but Ruiz et al*.* and Shin et al*.* had markedly younger participants of about 35 to 40 years [11, 15]. However, there is no single, easily distinguishable parameter—like age, sex, number of participants, group definition, extent of weight loss, or duration of intervention—in previous studies explaining why some did find different developments between MHO and MUO and others did not. Nevertheless, we were able to address several factors that might have influenced the discrepancies in the existing results: these include time points of measurements, group definition, and techniques of measuring metabolic changes. They will be discussed in the following paragraphs.

First, it is important to notice that in all previous studies only short-term effects were addressed, as the overall observation periods spanned from 3 to 9 months and measurements took place directly after the intervention, while we were able to track participants in our study for up to 18 months after weight loss (i.e. 21 months after study begin). Our study design allows separation of short-term and long-term effects of weight loss by repeated measurements after the weight loss intervention, which has not been done before. Thereby, we could confirm the studies demonstrating stronger short-term effects in MUO in several metabolic parameters, although both subgroups did benefit from weight loss. The between-group difference partly persisted over time during weight maintenance, which has not been reported before. Actually, most metabolic improvements dissolved in MHO while they were largely preserved in MUO. It cannot be ruled out that the disappearance of the metabolic benefits in MHO in the long term after weight loss results from ‘healthier’ baseline values compared to MUO. In a study on obese subjects, Klöting et al*.* found that insulin sensitive obese had a lower visceral fat area, reduced macrophage infiltration into the omental adipose tissue relevantly changing its structure as well as lower plasma levels of inflammatory parameters compared to insulin resistant obese subjects [7]. Hence, diverging constitutions of the adipose tissue might account for differences in the metabolic improvement between MHO and MUO and might be in the focus of future research.

Second, different definitions of metabolic health have been used in previous studies: in both studies that found no difference in the magnitude of metabolic change between MHO and MUO, group allocation followed the absence or presence of metabolic syndrome, respectively [11, 12]. In the other studies, group definition was done according to one of the following: absence or presence of metabolic syndrome [7, 15], presence of insulin resistance measured by oral glucose tolerance test [8] or hyperinsulinemic-euglycemic clamp [7, 9]. However, it remained unclear, whether the differential findings in previous studies are caused by the specific MHO/MUO definition used in each trial [7–9, 11, 12, 15]. We present data with three different group allocation definitions (by presence of metabolic syndrome (IDF definition), by HOMA-IR, and by ISI_Clamp_). On the whole, the results of all used classifications correspond well, confirming the findings of Janiszewski et al. who also used and compared two definition systems [7]. Nevertheless, in our study, differences between MUO and MHO appear to be partially greater in those parameters that were used for group allocation. While MUO classified by HOMA-IR or ISI_Clamp_ showed benefits preferentially in the glucose metabolism, the IDF classification revealed different benefits in MUO within the glucose and lipid metabolism as well. Overall however, the similarities in the results of both classifications outweigh the differences—especially regarding QoL, the groupings generally allow the same conclusion. So, group allocation by the highly sensitive, but vastly impractical hyperinsulinemic-euglycemic clamp is not necessary in clinical practice.

Last, most previous studies (including both studies that found no difference between the groups) reported metabolic changes in the lipid profile and the HOMA-IR as a measure of insulin sensitivity [8, 11, 12, 15] while a few reported results from the hyperinsulinemic-euglycemic clamp [7, 9] or an oral glucose tolerance test [8]. We are the first to report data from the hyperinsulinemic-euglycemic clamp as well as HOMA when comparing MHO and MUO regarding differences after weight loss. Interestingly, our data from the hyperinsulinemic-euglycemic clamp indicated a comparable improvement of the ISI_Clamp_ in both groups up to 12 months after weight loss. Currently, we have no definite explanation for this observation. However, the ISI_Clamp_ preferentially reflects myocellular insulin sensitivity. Hence, this might indicate that the differences in the weight loss-induced changes of HOMA-IR between MUO and MHO could rather be caused by changes in other body compartments than muscle, such as liver or subcutaneous fatty tissue. The use of labelled glucose for the hyperinsulinemic-euglycemic clamp in future studies could target and potentially answer this question [22]. For the first time, we report data on differences in QoL between MHO and MUO after weight loss.

MUO demonstrated a stronger and prolonged benefit in QoL, particularly in physical components of health. By definition, MUO have a higher number of comorbidities than MHO—at least when it comes to metabolic disorders. It is likely that the strong improvement of metabolic parameters in MUO contributes not only to amelioration of comorbidities but consequently also to improved physical health. The fact that MHO showed only short-term improvements in metabolism and QoL, while MUO demonstrated long-term improvements in both, allows the interpretation that improvement of metabolic parameters may be associated with improved physical health. Our data suggests that mental health is not as closely linked to weight and weight loss as physical health. In the long term, mental health was not improved irrespective of metabolic status. The predominant effect of weight loss on physical health is in line with results from other lifestyle intervention trials. In the Look AHEAD trial, the lifestyle intervention led to an improved physical but not mental function in the SF-36 over the course of the study [23]. Actually, the Look AHEAD trial included only overweight and obese subjects already diagnosed with type 2 diabetes. It makes this study population to some extent comparable to our metabolically ill MUO subgroup, although a preexisting diabetes was not mandatory in our MUO subjects. However, data regarding the effect of an identical weight loss/weight maintenance intervention on estimates of QoL in MUO and MHO has currently not yet been available, even if QoL has developed into an important outcome measure of weight loss therapies in general [24, 25]. It is of particular relevance for real-world settings, where changes in physical function strongly affect daily life and will therefore result in improved well-being. Moreover, intended improvement of QoL can be easily communicated to patients and will represent a substantial motivation to participate in weight loss programs. Therefore, our data gives new insight in the weight loss-induced long-term benefits in different obesity subgroups. Interestingly, these effects could apparently not be explained by differences in body weight changes, as both groups demonstrated a comparable weight course throughout the study.

Taken together, our data implies that MUO will benefit stronger in short- and long-term from weight loss interventions regarding several health-related outcomes. Thus, identification of obese patients with metabolic impairments might be a crucial strategy to increase the general efficacy of weight loss interventions in the context of health benefits. Although this was not directly addressed by our study, this assumption is supported by data from a previous meta-analysis investigating the effect of weight loss on all-cause mortality: Harrington et al*.* have shown that unhealthy obese lower their all-cause mortality by intentional weight loss while healthy obese appear to have an unaltered all-cause mortality after weight loss [26]. It is also in line with results of a large French cohort study that recently demonstrated a substantially higher risk for cardiovascular events in MUO compared to MHO [27]. However, most of the beneficial metabolic long-term effects in MUO shown in our study marginally failed to be significant in inter-group comparisons to MUO. This might be caused by the limited sample size and the high variability of individual long-term changes.

Interestingly, adjustment for improvement of different estimates of obesity during weight loss indicated that additional mechanisms apart from changes in body weight appear to play a relevant role for improvement of insulin sensitivity during weight loss in MUO. This might include inflammatory, metabolic, or hormonal factors such as leptin [28], adiponectin [28], or atrial natriuretic peptides [16]. Future research investigating the underlying mechanism of the stronger improvement of insulin resistance in MUO is warranted.

Our study comes with obvious limitations. First, the primary aim of the presented MAINTAIN trial was the comparison of different treatment strategies to achieve body weight maintenance. Thus, we defined the groups for this study retrospectively. However, MUO and MHO underwent the same procedures and we implemented potential relevant confounders including the treatment group in our statistical analyses. Participants in our trial were predominantly female, a distribution frequently found in lifestyle intervention trials. Although we statistically considered sex as an interfering factor in our linear model, we cannot rule out a bias by the sex imbalance. Furthermore, behavioral factors were not considered in this analysis, even though they are known to influence the long-term outcome of weight loss interventions [29]. Moreover, our trial suffered from a limited number of dropouts so that after 21 months only 112 from the original 143 participants could be analyzed. Unfortunately, only general reasons for dropout from this study were enquired. However, dropouts from a lifestyle intervention trial including a placebo group are not unexpected. A comparison of weight loss induced changes of metabolic and anthropometric parameters revealed comparable improvement in dropouts and subjects who completed the study (data not shown). We present data regarding obesity, metabolism, and clinical relevant outcomes like QoL. Nevertheless, effects on cardiovascular morbidity and mortality were out of scope of this study as it was primarily conceived to analyze anthropometric and metabolic outcomes after a lifestyle intervention. The strengths of our study include the large sample size with a long observation period, especially compared to previous studies. On top of that, we present data from comprehensive phenotyping at several time-points including the assessment of insulin sensitivity by the hyperinsulinemic-euglycemic clamp as well as highly relevant patient-reported outcomes of QoL. Additionally, we present data employing and comparing three different definitions for metabolic health.

## Conclusions

Overall, our data demonstrate that MUO have a greater benefit in health-related QoL and several metabolic parameters than MHO not only in the short-term but also in the long-term course after weight loss. However, it is necessary to remember that MHO still have increased rates of incident diabetes, heart failure, and mortality compared to metabolically healthy non-obese [30]. There might still be benefits from weight loss interventions regarding effects on the musculoskeletal system, among others, which were not addressed in this study. A recent review on this subject also points out that metabolically healthy obesity might represent a transitional state for some patients that might turn into metabolically unhealthy obesity at one point [31]. Thus, lifestyle modification should still be recommended for obese patients in general. In settings of limited resources the MUO might however become the preferential target group for lifestyle interventions.

## Supplementary Information

**Additional file 1.** Electronic supplementary material.

## Data Availability

The datasets generated during and/or analyzed during the current study are available from the corresponding author on reasonable request.

## Abbreviations

BMI: Body mass index
HbA1c: Hemoglobin A1c
HDL: High-density lipoprotein
HOMA-IR: Homeostatic model assessment for insulin resistance
IDF: Internationale Diabetes Federation
ISI_Clamp_: Insulin sensitivity index (hyperinsulinemic-euglycemic clamp)
LDL: Low-density lipoprotein
MHO: Metabolically healthy obese
MUO: Metabolically unhealthy obese
OGTT: Oral glucose tolerance test
QoL: Quality of life
SF-36: Short Form (36) Health Survey
WHO: World Health Organization
