# Supplementary material for: Long-term impact of the metabolic status on weight loss-induced health benefits
Source: Nutr Metab (Lond). 2022 Mar 28;19:25. doi: 10.1186/s12986-022-00660-w (PMC8962471; doi:10.1186/s12986-022-00660-w)
Supplement: Supplementary file 1 — Additional file 1. Electronic supplementary material. [file 12986_2022_660_MOESM1_ESM.pdf]

Supplemental Material

Long-term impact of the metabolic status on weight loss-induced health benefits

Dominik Soll[1], Julia Gawron[1], Laura Pletsch-Borba[1,4], Joachim Spranger[1,2,3,4], Knut Mai[1,2,3,4]

[1]Charité – Universitätsmedizin Berlin, corporate member of Freie Universität Berlin, Humboldt-Universität zu Berlin, and Berlin Institute of Health; Department of Endocrinology and Metabolism; 10117 Berlin, Germany.

[2]Charité – Universitätsmedizin Berlin, corporate member of Freie Universität Berlin, Humboldt-Universität zu Berlin, and Berlin Institute of Health; Charité Center for Cardiovascular Research; 10117 Berlin, Germany.

[3]DZHK (German Centre for Cardiovascular Research), partner site Berlin

[4]NutriAct-Competence Cluster Nutrition Research Berlin-Potsdam

1. Pre-trial weight loss phase:

The protocol of the 12 weeks' weight loss program included three components: caloric restriction, nutritional counseling and physical exercises. Caloric restriction was applied during the weight reduction program in two stages. A replacement of all 3 meals by a very-low energy diet (Optifast 2®, Nestlé HealthCare Nutrition GmbH, Frankfurt am Main, Germany) was performed during the first eight weeks. This diet supplied 800 kcal per day and was provided by the trial team for free. Participants received 35 portions of formula diet for each week (5 per day). The participants were advised not to consume any additional food. After 8 weeks the diet was switched to an energy-reduced healthy diet composed as a balanced mix (carbohydrates 35-45 %, fat 25-35 %, and protein 25-30 %). Although daily calorie intake of approximately 1500 kcal was recommended, individual counseling was performed based on information of the initial eating protocols, measured energy expenditure and reported physical activity.

Weekly meetings including dietary advices for healthy living, group workshops with practical cooking exercises and recommendation regarding increased physical activity were performed during the entire weight loss period of 12 weeks. Psychologists also attended the workshops during four dates at week 5, 7, 9 and 11. A physician gave medical advice during one of the meetings as well. Compliance of the diet was supported by providing specific recipes, cooking advices, and instructions for behavior modifications (only 3 meals per day, at least 4 hours break between the meals, reduced carbohydrate intake at dinner). In the first two months, weight loss and health status were monitored weekly, which included a patient interview and blood pressure measurement. Body weight was measured at least once per week. A supervised 30 min exercise session was performed after each meeting to support the recommended increase of physical activity. Participants were also encouraged to attend at least one additional physical exercise course per week.

2. 12-months randomized weight maintenance phase:

Subjects who lost at least 8% of their body weight during the weight loss phase (n=143) were randomized into an intervention and a control group. Subjects in the control group received an advice leaflet and were asked to return for examination after 12 and 18 months. However, they were no longer involved in any form of counseling. Continuous counseling was performed in the intervention group for the next 12 months in gradually diminishing frequency. Weekly group sessions, comparable to sessions of the weight loss period, were performed for the first sixteen weeks of the weight maintenance period. Subsequently meetings were taking place each two weeks over a period of two months. Afterwards monthly meetings were performed until the end of the intervention period. Thus, at least 36 meetings were offered every participants of the intervention group during 12 months weight maintenance phase. The dietary advices within the intervention group were focused on a balanced diet advocating the preferential intake of specific foods (like high intake of vegetables, cereals, fat reduced foods, lean meat consumption (lean fish and chicken)). The recommended distribution of macronutrients (35-45% carbohydrates, 25-35% fat, and 25-30% protein) was comparable to the final phase of the weight loss intervention. An individual caloric intake was calculated and further adapted to achieve body weight maintenance. Therefore, body weight was measured during every group session.

The supervised physical activity regime was maintained for the first 12 weeks of weight maintenance period. Thereafter participants were encouraged to exercise at least twice a week but without direct supervision. To increase motivation and allow self-monitoring, pedometers were given to the participants and a gym membership was offered. The mentioned psychological support was continued for 6 additional dates.

3. Legends of Supplementary Tables and Figures

Table S1. Estimates of clinical parameters of all participants before (T_{-3}) and after (T_0) weight loss as well as after 12 months of weight maintenance (T_{12}) and additional 6 months of follow-up (T_{18}).

Values shown (except for n, randomization, and age) are estimated marginal means with 95% confidence intervals from model without further adjustments.

* $p < 0.05$, ** $p < 0.01$, *** $p < 0.001$ vs. T_{-3}

Table S2. Estimates of clinical parameters of MHO/MUO as defined by low/high HOMA-IR.

Values shown (except for n, randomization, and age) are estimated marginal means with 95% confidence intervals from model with adjustment to sex, age, and randomisation.

* $p < 0.05$, ** $p < 0.01$, *** $p < 0.001$ vs. T_{-3} in respective group

Table S3. Estimates of clinical parameters of MHO/MUO as defined by high/low ISI_{Clamp}.

Values shown (except for n, randomization, and age) are estimated marginal means with 95% confidence intervals from model with adjustment to sex, age, and randomisation.

* $p < 0.05$, ** $p < 0.01$, *** $p < 0.001$ vs. T_{-3} in respective group

Figure S1. Flow chart of the randomized controlled trial (MAINTAIN trial).

Figure S2. Effect of weight loss and maintenance on insulin resistance (adjusted for waist circumference (A) or fat mass (B)) in MHO/MUO.

MHO + MUO were assigned following the IDF criteria for metabolic syndrome. Values represent estimated marginal means with 95% confidence intervals from model with adjustment to sex, age, randomisation, and waist circumference [in cm] or fat mass [in kg] at all time-points.

$^{*} p < 0.05$, $^{**} p < 0.01$, $^{***} p < 0.001$ vs. T_{-3} in respective group

4. Supplementary Tables

Table S1.

	T_{-3}	T_0	T_{12}	T_{18}
n: m/w	143: 31/112	143: 31/112	121: 26/95	112: 25/87
Randomization: control/intervention	71/72			
Age [years]	50.5 [48.5,52.6]			
BMI [kg/m2]	37.3 [36.4,38.3]	32.7 [31.7,33.7]***	33.4 [32.4,34.4]***	34.4 [33.4,35.4]***
Waist circumference [cm]	108.2 [106.1,110.3]	98.8 [96.7,100.9]***	99.7 [97.6,101.9]***	100.8 [98.6,103]***
Serum glucose [mg/dl]	89.9 [87.6,92.2]	84.1 [81.8,86.4]***	88.2 [85.7,90.7]	91.5 [89,94]
HbA1c [%]	5.8 [5.7,5.9]	5.9 [5.8,6]	5.6 [5.4,5.7]**	5.7 [5.6,5.8]
HOMA-IR	2.9 [2.6,3.2]	1.6 [1.3,1.9]***	1.9 [1.6,2.2]***	2.3 [2,2.6]**
ISI_{Clamp} [mg•kg^{-1}•min^{-1}/(mU•l^{-1})]	0.06 [0.06,0.07]	0.09 [0.08,0.09]***	0.08 [0.08,0.09]***	
Total cholesterol [mg/dl]	203.6 [197.2,210]	173.8 [167.4, 180.2]***	196.3 [189.6,203]*	198.1 [191.3,204.9]
LDL-Cholesterol [mg/dl]	124.8 [119.5,130.2]	106.3 [101,111.6]***	118.1 [112.5,123.7]*	122.4 [116.7,128]
HDL-Cholesterol [mg/dl]	52 [49.7,54.4]	47.8 [45.5,50.2]***	56.7 [54.2,59.2]***	56.4 [53.9,58.9]***
Triglycerides [mg/dl]	142 [128.2,155.7]	93.5 [79.7,107.2]***	118.3 [103.5,133.2]*	114.5 [99.3,129.6]**
Systolic RR [mmHg]	129.3 [126,132.5]	119.2 [116,122.4]***	122.7 [119.3,126.1]*	124.6 [121,128.2]
Diastolic RR [mmHg]	80.9 [79.2,82.6]	74.9 [73.3,76.6]***	79 [77.2,80.7]	79.2 [77.4,81.1]

Table S2.

	Metabolically Healthy Obese (MHO)				Metabolically Unhealthy Obese (MUO)				p-value (MHO vs MUO)		
	T_{-3}	T_0	T_{12}	T_{18}	T_{-3}	T_0	T_{12}	T_{18}	$T_{-3} \rightarrow T_0$	$T_{-3} \rightarrow T_{12}$	$T_{-3} \rightarrow T_{18}$
n: m/w	71: 10/61	71: 10/61	62: 10/52	58: 9/49	71: 21/50	71: 21/50	58: 16/42	53: 16/37			
Randomization: control/intervention	31/40				40/31						
Age [years]	51.7 [48.7,54.6]				49.4 [46.4, 52.4]						
BMI [kg/m2]	35.4 [33.8,36.9]	31.1 [29.5,32.7]***	31.8 [30.2,33.4]***	33 [31.4,34.6]***	39.1 [37.6,40.5]	34 [32.6,35.5]***	34.9 [33.5,36.4]***	35.7 [34.3,37.2]***	0.31	0.92	0.18
Waist circumference [cm]	107.2 [104.1,110.3]	98.4 [95.3,101.4]***	99.2 [96,102.3]***	100.7 [97.5,103.9]***	115.2 [112.4,118]	105.3 [102.5,108.1]***	106.2 [103.3,109]***	106.7 [103.7,109.6]***	1	1	0.93
Serum glucose [mg/dl]	83.6 [80.3,87]	80.9 [77.6,84.3]	83.8 [80.3,87.3]	87.4 [83.8,90.9]	95.6 [92.5,98.8]	86.8 [83.6,89.9]***	90.7 [87.4,94.1]*	93.5 [90,96.9]	0.03	0.18	0.09
HbA1c [%]	5.6 [5.4,5.7]	5.8 [5.6,6]*	5.4 [5.2,5.6]	5.5 [5.4,5.7]	6 [5.8,6.1]	6 [5.8,6.1]	5.6 [5.4,5.8]**	5.7 [5.5,5.9]	0.54	1	0.79
HOMA-IR	1.6 [1.2,2]	1.4 [0.9,1.8]	1.5 [1.1,2]	1.8 [1.4,2.2]	4.5 [4.1,4.9]	2.1 [1.8,2.5]***	2.5 [2.1,3]***	3.1 [2.6,3.5]***	<0.001	<0.001	<0.001
ISI_{Clamp} [$mg \cdot kg^{-1} \cdot min^{-1}/(mU \cdot l^{-1})$]	0.08 [0.07,0.09]	0.10 [0.10,0.11]***	0.10 [0.09,0.11]**		0.04 [0.03,0.05]	0.07 [0.06,0.08]***	0.07 [0.06,0.08]***		1	0.17	
Total cholesterol [mg/dl]	204 [194.3,213.8]	170.7 [160.9,180.4]***	194.9 [184.8,205]	195.1 [184.9,205.3]	194.6 [185.6,203.6]	168.1 [159.2,177.1]***	185.8 [176.3,195.4]	188.4 [178.8,198.1]	0.99	1	1
LDL-Cholesterol [mg/dl]	125.8 [117.3,134.2]	103.4 [95,111.8]***	116.1 [107.4,124.9]*	119.8 [111,128.6]	117.8 [110,125.5]	103 [95.3, 110.8]***	112 [103.9,120.2]	116.5 [108.2,124.8]	0.39	1	1
HDL-Cholesterol [mg/dl]	51.9 [48.4,55.4]	46.7 [43.2,50.3]***	55.9 [52.3,59.6]**	56.1 [52.4,59.7]**	46.1 [42.8,49.3]	42.6 [39.3,45.8]**	50.6 [47.2,54]***	49.5 [46.1,52.9]*	1	1	1
Triglycerides [mg/dl]	140.8 [119.5,162]	102.2 [80.9,123.5]***	123.7 [101.2,146.3]	115 [92.1,137.8]	167.3 [147.5,187.2]	108.7 [88.9,128.6]***	134.4 [112.7,156.1]*	134.8 [112.5,157]*	0.94	1	1
Systolic RR [mmHg]	128.5 [123.7,133.3]	119.6 [114.7,124.4]*	121.8 [116.8,126.9]	120.4 [115.2,125.7]	132.3 [127.6,136.9]	120.8 [116.3,125.4]***	124.6 [119.6,129.6]	129.6 [124.4,134.9]	1	1	1
Diastolic RR [mmHg]	80.4 [77.7,83]	74.7 [72,77.3]***	78.7 [75.9,81.4]	79 [76.1,81.8]	80.6 [78.1,83.1]	74.3 [71.8,76.7]***	78.4 [75.8,81.1]	78.4 [75.7,81.2]	1	1	1

Table S3.

	Metabolically Healthy Obese (MHO)				Metabolically Unhealthy Obese (MUO)				p-value (MHO vs MUO)		
	T_{-3}	T_0	T_{12}	T_{18}	T_{-3}	T_0	T_{12}	T_{18}	$T_{-3} \rightarrow T_0$	$T_{-3} \rightarrow T_{12}$	$T_{-3} \rightarrow T_{18}$
n: m/w	70: 11/59	70: 11/59	63: 11/52	58: 10/48	69: 18/51	69: 18/51	55: 13/42	50: 13/37			
Randomization: control/intervention	35/35				35/34						
Age [years]	49.9 (47.1, 52.7)				50,9 (47.7, 54.2)						
BMI [kg/m2]	34.5 (33, 36.1)	30.2 (28.7, 31.8)***	31.5 (29.9, 33)***	32.4 (30.8, 33.9)***	39.7 (38.3, 41.2)	34.8 (33.4, 36.2)***	35.2 (33.8, 36.7)***	36.3 (34.9, 37.8)***	0.59	0.005	0.03
Waist circumference [cm]	106.1 (103.2, 109)	96.6 (93.7, 99.5)***	98.7 (95.7, 101.6)***	99.8 (96.8, 102.8)***	116.5 (113.8, 119.3)	107.1 (104.4, 109.9)***	106.7 (103.9, 109.6)***	107.3 (104.4, 110.2)***	1	0.43	0.2
Serum glucose [mg/dl]	85.8 (82.3, 89.3)	82.2 (78.7, 85.7)	86.1 (82.5, 89.7)	89.3 (85.7, 93)	94.7 (91.4, 98)	86.5 (83.1, 89.8)***	89.5 (85.9, 93.1)*	92.8 (89.1, 96.5)	0.24	0.13	0.16
HbA1c [%]	5.6 (5.4, 5.8)	5.9 (5.7, 6.1)**	5.5 (5.3, 5.7)	5.5 (5.3, 5.7)	6 (5.8, 6.1)	5.9 (5.7, 6.1)	5.6 (5.4, 5.8)***	5.8 (5.6, 6)	0.03	0.53	1
HOMA-IR	2 (1.6, 2.4)	1.4 (1, 1.9)*	1.9 (1.4, 2.3)	2 (1.6, 2.5)	4.2 (3.8, 4.6)	2.2 (1.8, 2.6)***	2.4 (1.9, 2.9)***	3 (2.6, 3.5)***	<0.001	<0.001	0.001
ISI_{Clamp} [$mg \cdot kg^{-1} \cdot min^{-1}/(mU \cdot l^{-1})$]	0.09 (0.08, 0.10)	0.11 (0.10, 0.12)***	0.10 (0.09, 0.11)**		0.04 (0.03, 0.05)	0.07 (0.06, 0.07)***	0.07 (0.06, 0.07)***		0.41	0.04	
Total cholesterol [mg/dl]	200.9 (191.1, 210.7)	167.7 (157.9, 177.5)***	189.7 (179.7, 199.8)*	191.1 (181, 201.3)	198.8 (189.5, 208)	169.9 (160.7, 179.2)***	191.6 (181.6, 201.5)	191.6 (181.6, 201.7)	1	1	1
LDL-Cholesterol [mg/dl]	122.5 (114.1, 131)	101 (92.6, 109.4)***	112.6 (104, 121.2)**	116.9 (108.2, 125.6)	121.7 (113.7, 129.7)	104.3 (96.3, 112.3)***	116.1 (107.6, 124.6)	118.7 (110.2, 127.3)	1	1	1
HDL-Cholesterol [mg/dl]	51.5 (48, 55)	46.4 (42.8, 49.9)***	55.1 (51.5, 58.7)**	55.1 (51.4, 58.7)*	45.8 (42.4, 49.1)	42.1 (38.8, 45.4)**	50.3 (46.9, 53.8)***	49.4 (45.9, 52.9)*	1	1	1
Triglycerides [mg/dl]	142.7 (121.5, 163.9)	102.4 (81.2, 123.6)***	124.1 (101.9, 146.3)	114 (91.4, 136.6)	171.1 (150.8, 191.4)	113 (92.6, 133.3)***	140.2 (117.6, 162.7)*	141.8 (119, 164.7)	1	1	1
Systolic RR [mmHg]	126.8 (121.9, 131.6)	118.2 (113.4, 123)*	121.3 (116.3, 126.2)	125.7 (120.5, 131)	133.7 (129, 138.4)	121.8 (117.2, 126.4)***	125.3 (120.2, 130.5)	124.3 (118.8, 129.7)*	1	1	0.39
Diastolic RR [mmHg]	78.8 (76.2, 81.5)	73.8 (71.2, 76.5)**	77.6 (74.9, 80.3)	78.2 (75.3, 81)	81.8 (79.3, 84.4)	74.9 (72.4, 77.4)***	79.2 (76.5, 81.9)	79.3 (76.4, 82.1)	1	1	1

Figure S1.

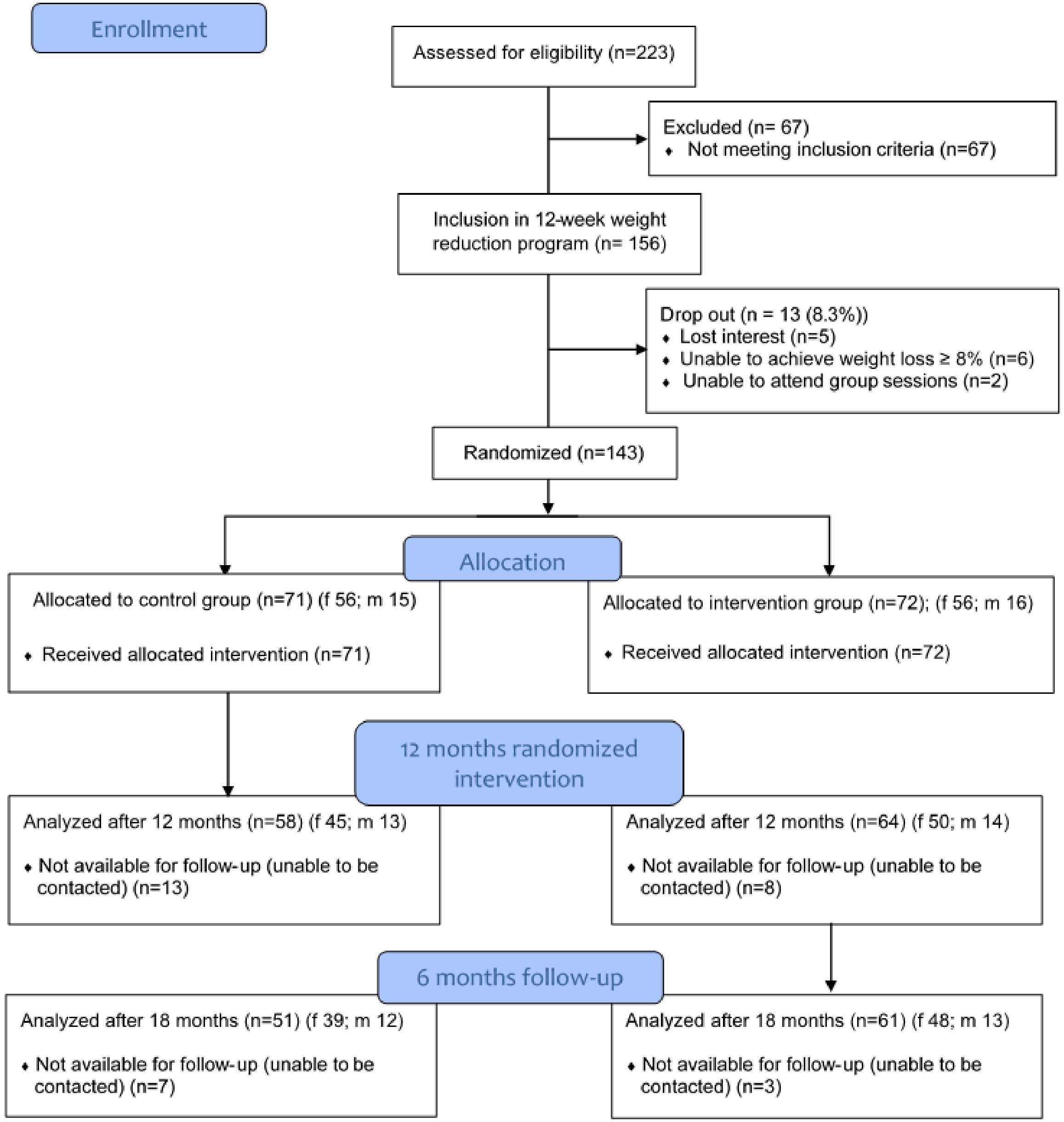
Enrollment
Assessed for eligibility (n=223)
Excluded (n= 67)
Not meeting inclusion criteria (n=67)
Inclusion in 12-week weight reduction program (n= 156)
Drop out (n = 13 (8.3%))
Lost interest (n=5)
Unable to achieve weight loss ≥ 8% (n=6)
Unable to attend group sessions (n=2)
Randomized (n=143)
Allocation
Allocated to control group (n=71) (f 56; m 15)
Received allocated intervention (n=71)
Allocated to intervention group (n=72); (f 56; m 16)
Received allocated intervention (n=72)
12 months randomized intervention
Analyzed after 12 months (n=58) (f 45; m 13)
Not available for follow-up (unable to be contacted) (n=13)
Analyzed after 12 months (n=64) (f 50; m 14)
Not available for follow-up (unable to be contacted) (n=8)
6 months follow-up
Analyzed after 18 months (n=51) (f 39; m 12)
Not available for follow-up (unable to be contacted) (n=7)
Analyzed after 18 months (n=61) (f 48; m 13)
Not available for follow-up (unable to be contacted) (n=3)

Figure S2.

A

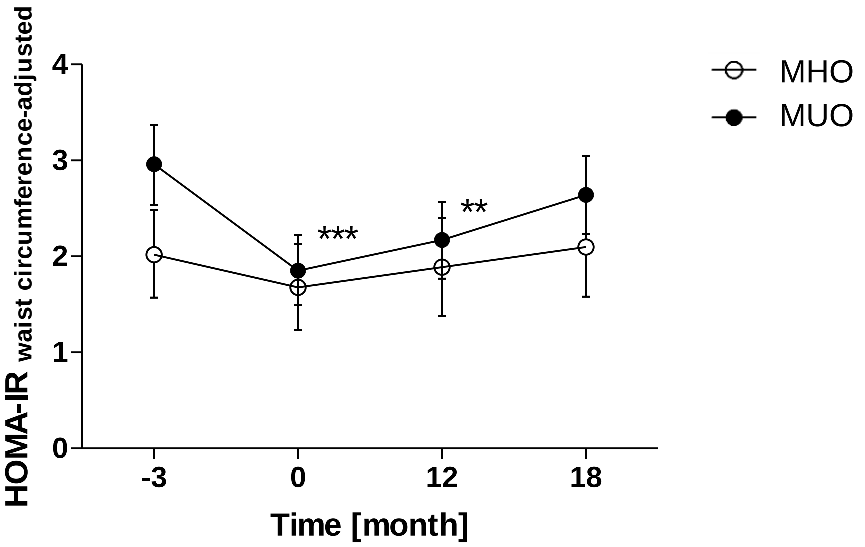
HOMA-IR waist circumference-adjusted
4
3
2
1
0
-3
0
12
18
Time [month]
***
**
MHO
MUO

B

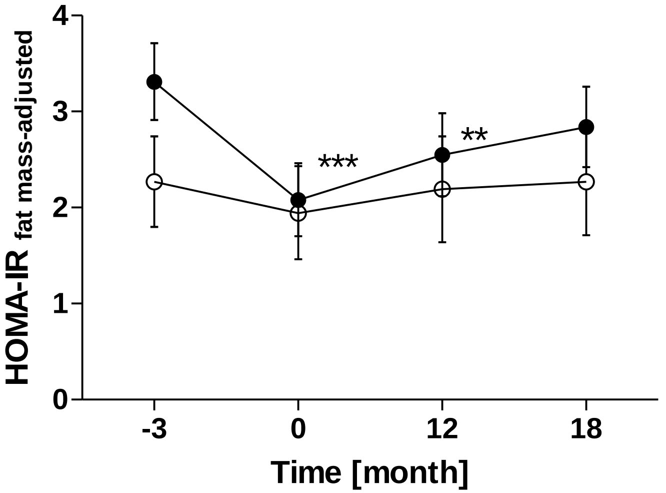
HOMA-IR fat mass-adjusted
4
3
2
1
0
-3
0
12
18
Time [month]
***
**
